# Supplementary material for: Facile Synthesis of Two Dimensional (2D) V2O5 Nanosheets Film towards Photodetectors
Source: Materials (Basel). 2022 Nov 23;15(23):8313. doi: 10.3390/ma15238313 (PMC9740591; doi:10.3390/ma15238313)
Supplement: Supplementary file 1 [file materials-15-08313-s001.zip › materials-2009499-supplementary.pdf]

Supplementary Materials:

Facile Synthesis of Two dimensional (2D) V_2O_5 Nanosheets Film towards Photodetectors

Shaotian Wang [1], Liangfei Wu [2], Hui Zhang [1], Zihan Wang [1], Qinggang Qin [1], Xi Wang [3], Yuan Lu [3], Liang Li [2] and Ming Li [2,*]

[1] Institutes of Physical Science and Information Technology, Anhui University, Hefei 230601, China
[2] Key Laboratory of Materials Physics, Anhui Key Laboratory of Nanomaterials and Nanotechnology, Institute of Solid State Physics, Hefei Institutes of Physical Science, Chinese Academy of Sciences, Hefei 230031, China
[3] State Key Laboratory of Pulsed Power Laser Technology, Anhui Laboratory of Advanced Laser Technology, Infrared and Low Temperature Plasma Key Laboratory of Anhui Province, National University of Defense Technology, Hefei 230037, China
* Correspondence: liming@issp.ac.cn

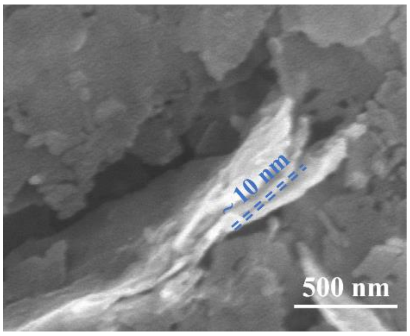

Figure S1. High-magnification SEM image of V_2O_5 nanosheets.

Table S1. Comparison of key parameters of V_2O_5 nanostructured photodetectors

Materials structure	Condition, wavelength	R (A W^{-1})	D* (Jones)	EQE (%)	Response time	Shape size	Ref.
V_2O_5 Nanosheets	5 V, 405 nm	0.0029	2.05×10^6	0.8773	2.4 s/4.7 s	Size＞500 nm, thickness~10 nm	This Work
	1 V, 400 nm	0.062	-	-	65 s/75 s	Size~tens of μm, thickness~50 nm	[1]
	1 V, 554 nm	0.020	-	-	＞10 s	Size~3 μm, thickness~200 nm	[2]
V_2O_5 Nanowires	0.1 V, 325 nm	7900	-	-	1000/2000 s	Diameter~60 nm, length＞10μm	[3]
	1 V, 532 nm	0.00065	-	-	9/10 s	Length~several μm, width~156 nm, thickness~24 nm	[4]
V_2O_5 Nanorods	1 V, 540 nm	0.984	-	-	0.914/0.573 s	Diameters~60-80 nm, length of 200 nm	[5]
	0.5 V, 530 nm	0.042	-	-	0.735/0.134 s	Diameters~75-120 nm, length of 200 nm	[6]

References

1. M. S. Pawar, P. K. Bankar, M. A. More and D. J. Late, *RSC Advances*, 2015, **5**, 88796-88804.
2. B. P. Yalagala, P. Sahatiya, C. s. R. Kolli, S. Khandelwal, V. Mattela and S. Badhulika, *ACS Applied Nano Materials*, 2019, **2**, 937-947.
3. R. S. Chen, W. C. Wang, C. H. Chan, H. P. Hsu, L. C. Tien and Y. J. Chen, *Nanoscale Res Lett*, 2013, **8**, 443.
4. J. Wu, S. Ding, Z. Huang, H. Li, K. Huang, X. Qi and J. Li, *Fullerenes, Nanotubes and Carbon Nanostructures*, 2019, **27**, 566-571.
5. N. M. Abd-Alghafour, N. M. Ahmed and Z. Hassan, *Sensors and Actuators A: Physical*, 2016, **250**, 250-257.
6. N. M. Abd-Alghafour, N. M. Ahmed, Z. Hassan and M. Bououdina, *Applied Physics A*, 2016, **122**.
